# The genome of the MZM-0403 strain of the African turquoise killifish, *Nothobranchius furzeri*

**DOI:** 10.1093/g3journal/jkaf075

**Published:** 2025-04-07

**Authors:** Bernadette D Johnson, Balan Ramesh, Adam G Jones

**Affiliations:** Department of Zoology and Biodiversity Research Centre, The University of British Columbia, Vancouver V6T 1Z4, British Columbia, Canada; Centre for Life Sciences, Mahindra University, Survey No: 62/1A, Bahadurpally Jeedimetla, Hyderabad, Telangana 500043, India; Department of Biological Sciences, University of Idaho, Moscow ID 83844-3051, USA

**Keywords:** aging, annotation, hifiasm, genomics, long-read sequencing, pacBio, whole-genome assembly, *Nothobranchius furzeri*, MZM-0403, Genome Assembly

## Abstract

The African turquoise killifish, *Nothobranchius furzeri*, is an emerging model for functional genomics research. Interest in *N. furzeri* stems from its extremely short lifespan, and the breadth of research on this fish is rapidly expanding. All currently available whole-genome assemblies for *N. furzeri* are based on the *GRZ* strain. However, *N. furzeri* shows substantial phenotypic differences among populations. Here, we present a whole-genome assembly of the MZM-0403 strain of *N. furzeri*, which differs from the *GRZ* strain with respect to lifespan and male coloration patterns. We used PacBio HiFi sequencing to sequence the genome of an MZM-0403 male to ∼48 × coverage. The PacBio reads were de novo assembled and then scaffolded against an existing *N. furzeri* genome assembly. This strategy resulted in a chromosome-level assembly. Our MZM-0403 assembly differs from previous *N. furzeri* assemblies in that it is closer to the expected genome size based on independent estimates (∼1.5 Gb) and it has substantially fewer gaps, particularly in the vicinity of genes and within introns. A repeat analysis shows that about two-thirds of the genome is composed of repetitive elements. In addition, our assembly approach allowed us to recover phased fragments of the X- and Y-chromosomes. Analysis of these regions identifies 20 genes that are likely in the nonrecombining region of the sex chromosomes. Overall, this novel genome assembly will be useful for future functional and comparative genomics studies of fishes in the genus *Nothobranchius*.

## Introduction

The African turquoise killifish, *Nothobranchius furzeri*, is an emerging model system for vertebrate functional genomics and molecular biology (Cellerino *et al*. 2016; Platzer and Englert 2016; Reichard and Polačik 2019). Much of the interest in this fish stems from its extremely short lifespan (Valdesalici and Cellerino 2003), which makes it an appealing model for ageing research. Some strains of *N. furzeri* have median lifespans ranging from 2 to 4 months (Valdesalici and Cellerino 2003; Terzibasi *et al*. 2008), giving this species the second-shortest documented vertebrate lifespan (behind the coral reef pygmy goby; Depczynski and Bellwood 2005) and the shortest for a vertebrate that can be readily maintained in a laboratory setting. While the short lifespan of *N. furzeri* makes it an appealing model for ageing research, its short generation time also makes it useful for a wide range of other research enterprises, so the breadth of work on this fish is rapidly expanding (e.g. Hu *et al*. 2020; Johnson *et al*. 2020; Giaquinto *et al*. 2024).

One important step toward establishing *N. furzeri* as a useful model was the sequencing of its genome. Two separate research groups published papers documenting whole-genome assemblies and annotations of the GRZ strain of *N. furzeri* in 2015 (Reichwald *et al*. 2015; Valenzano *et al*. 2015). The GRZ strain is the most-used laboratory strain with the shortest lifespan of the various strains that have become established for laboratory-based research (Terzibasi *et al*. 2008). This strain was first collected from temporary ponds in the Gonarezhou National Park of Zimbabwe in 1968 (Jubb 1971), and it has been maintained in captivity since that time. Consequently, it is highly homozygous, a feature that facilitates genome assembly. Recently, Willemsen *et al*. (2020) produced an updated genome assembly for the GRZ strain by integrating existing data from the first two assemblies with some newly generated sequence data. At present, then, 3 genome assemblies for the GRZ strain of *N. furzeri* are published and publicly available (NCBI accession numbers GCA_001465895.2, GCA_000878545.1, and GCA_014300015.1).

The 3 current assemblies for GRZ have been extremely useful for various research enterprises. One positive aspect of these assemblies is that they are fully annotated and the gene content is extremely well represented. The Reichwald *et al*. (2015) and Willemsen *et al*. (2020) assemblies also contain chromosome-level scaffolds, recovering the haploid count of 19 chromosomes for *N. furzeri*. However, these assemblies also have some disadvantages, which were unavoidable with the technology available at the time they were produced. For instance, outside of the coding sequences, they contain many gaps, as evidenced by the Willemsen *et al*. (2020) assembly, which has a scaffold N50 of 49.9 Mb (according to NCBI), but a contig N50 of only 26 kb. In addition, the assemblies, at around 1–1.25 billion base pairs in total length, are much smaller than independent estimates of *N. furzeri* genome size, which suggest a genome on the order of 1.5 billion base pairs. This discrepancy is due to a lack of representation of repetitive sequences in the assemblies (Reichwald *et al*. 2015). In short, these genomes, with their excellent coverage of gene content, are useful for many applications, but they still have substantial room for improvement given recent advances in sequencing technology.

Our goal here is to contribute to the development of genomic resources in *N. furzeri* by generating a nearly complete genome assembly of another strain, *MZM-0403*, that is phenotypically distinct in several ways from the previously sequenced *GRZ* strain. The *MZM-0403* strain was collected in 2004 from a site near the Limpopo River in Mozambique (Terzibazi *et al*. 2008). The most prominent phenotypic differences between the *GRZ* and *MZM-0403* strains include the tail coloration of males—yellow in the former and red in the latter—and the considerably longer lifespan of MZM-0403 individuals compared to those of the GRZ strain. For instance, Terzibasi *et al*. (2008) reported a median lifespan of 23 weeks for MZM-0403 fish compared to a median lifespan of only around 9 weeks for members of the GRZ strain. While QTL mapping studies have attempted to identify loci underlying lifespan and male tail coloration differences among strains (Valenzano *et al*. 2009, 2015; Kirschner *et al*. 2012), researchers have not yet pinpointed the causative loci associated with these divergent phenotypes. These genome assemblies have, however, confirmed that *N. furzeri* has an XY sex-determination system, and they establish this species as an interesting model for the study of the evolution of sex chromosomes (Reichwald *et al*. 2015; Valenzano *et al*. 2015; Štundlová *et al*. 2022).

Here, we report the whole-genome sequencing of the MZM-0403 strain of *N. furzeri*. Our primary aim is to provide a resource for future comparative studies among strains that display interesting patterns of trait divergence. Our sequencing also reveals some deficiencies in the current GRZ genome assemblies, as well as some intriguing features of the sex chromosomes. This new genomic resource will facilitate future research on this increasingly popular emerging model system.

## Methods

### Sequencing, assembly, and annotation

We obtained a single, lab-reared, adult male MZM-0403 *N. furzeri* specimen from Dr Chi-Kuo Hu at Stony Brook University. This specimen had been flash frozen in liquid nitrogen and was shipped to the University of Idaho on dry ice. We used the QIAgen MagAttract HMW DNA Kit to extract high molecular weight DNA from brain and eye tissue from this single adult male. The DNA was transported to the University of Idaho Institute for Interdisciplinary Data Sciences Genomics and Bioinformatics Resources Core, where a PacBio HiFi SMRTbell library was prepared using the SMRTbell Express Template Prep Kit 2.0. These libraries were then sequenced using 4 PacBio Sequel II SMRTcells.

PacBio HiFi reads were assembled into contigs using Hifiasm v. 0.16.1-r375 (Cheng *et al*. 2021, 2022, 2024). The consensus primary contigs produced by Hifiasm were then scaffolded against the 2015 GRZ assembly (Reichwald *et al*. 2015; GCF_001465895.1) using ntJoin v. 1.0.8 (Coombe *et al*. 2020) with 500 base pairs as the window size and the default k-mer size of 32. We chose to use the GRZ_2015 assembly rather than the 2020 reassembly by Willemsen *et al*. (2020), because the 2015 assembly integrated optical mapping data into their scaffolding procedure, whereas the 2020 assembly did not. In addition, the 2015 assembly used DNA from a female, and the 2020 assembly used male data. Because *N. furzeri* has an XY sex-determination system (Reichwald *et al*. 2015; Valenzano *et al*. 2015), with X- and Y-chromosomes that appear not to be strongly differentiated in terms of gene content (Štundlová *et al*. 2022), the female-specific assembly is more likely to provide a correctly scaffolded X-chromosome.

The final MZM-0403 assembly was uploaded to NCBI (accession number GCF_027789165.1) and annotated via the NCBI Eukaryotic Genome Annotation Pipeline. The complete genome sequences and annotation files are currently publicly available.

### Quality assessment and comparison to existing assemblies

We used Assembly Stats v1.0.1 (Trizna 2020) and BUSCO v5.7.1 (Manni *et al*. 2021) to compare our new MZM-0403 assembly to the 2015 GRZ assembly of Reichwald *et al*. (2015) and the 2020 GRZ assembly of Willemsen *et al*. (2020), hereafter referred to as GRZ_2015 and GRZ_2020, respectively. For the BUSCO analysis, we used the Actinopterygii odb10 database and the “−augustus” parameter to specify Augustus (Stanke and Waack 2003; Keller *et al*. 2011) as the algorithm for gene prediction.

We used MCscanX v1.0.0 (Wang *et al*. 2012) to compare the order of genes on the chromosomes of the different assemblies. We used the default settings for MCscanX, and performed reciprocal blastp searches among all annotated protein-coding sequences from each of the 3 genomes to identify similar gene products within and between assemblies. MCscanX results were plotted using SynVisio (Bandi and Gutwin 2020). To visualize chromosome-level similarities and differences among the assemblies, we also produced dot plots between homologous chromosomes using the online tool D-Genies (Cabanettes and Klopp 2018) under default settings.

### Repeat analysis

We used RepeatMasker version 4.1.7-p1 to characterize repetitive DNA elements in our MZM assembly. This analysis was conducted using default settings, and we used the *N. furzeri* repeat library “Nf-RepLib.20141117.fa” as our repeat reference database (Reichwald *et al*. 2015). This repeat database can be downloaded from the following website: https://nfingb.leibniz-fli.de/.

### Sex chromosome fragments

Since Hifiasm produces phased haplotypes during its initial contig assembly phase, we anticipated that phased contigs would include fragments of the X- and Y-chromosomes. In addition to the consensus primary assembly, Hifiasm produces 2 fully phased assemblies (hap1 and hap2), representing the 2 different chromosome copies for the diploid individual from which DNA was obtained and sequenced. We focused on these phased assemblies (i.e. hap1 and hap2) in our search for the sex chromosomes.

Previous work identified the locus *gdf6* as the most likely candidate for the sex-determining gene (Reichwald *et al*. 2015). We obtained the *N. furzeri gdf6* coding sequence from NCBI and used blastn to search for this locus in the hap1 and hap2 assemblies. One contig from each phased assembly displayed a significant hit in our *gdf6* blast search. By comparing the sequences of *gdf6* on these contigs to the known sequences of *gdf6* from the X- and Y-chromosomes of *N. furzeri*, we established that contig “h1tg000299l” from the hap1 assembly represented a 3,385,904 bp fragment of the X-chromosome, and contig “h2tg000474l” was a 4,453,144 bp fragment of the Y-chromosome.

We compared these X- and Y-chromosome fragments to each other and to the GRZ_2015 reference genome by using a combination of dot plots and RepeatMasker. We also used a custom blast strategy to search for genes that are known from the GRZ_2015 assembly to occur upstream and downstream of the *gdf6* locus. We aligned and compared these genes between the X- and Y-chromosomal fragments and to the GRZ_2015 annotation to examine sequence divergence in this region.

## Results and discussion

### Sequencing, assembly, and annotation

We generated 10,423,972 PacBio HiFi reads, which amounted to a total of 72,541,030,864 base pairs of sequence data. Based on an estimated genome size of 1.5 billion base pairs for *N. furzeri* (Reichwald *et al*. 2009, 2015), this dataset provided 48 × coverage. The mean read size was 6,959.06 base pairs, the longest read was 44,789 base pairs, and the raw read N50 was 7,436 (*n* = 3,792,401).

The initial Hifiasm assembly of our MZM-0403 data produced 2,232 contigs, with a total length of 1.40 billion base pairs (Table 1). The N50 of this initial assembly was 1,564,885 base pairs (*n* = 247), and the N90 was 390,456 base pairs (*n* = 922). The contigs contained no gaps or unknown base pairs (i.e. Ns). After scaffolding with ntJoin against the 2015 GRZ genome assembly, the length of our MZM-0403 assembly increased to 1.51 billion base pairs. This increase in length resulted from the addition of 3,093 gaps, which added a total of 102,568,068 unknown base pairs to the scaffolds (Table 1). The final MZM-0403 assembly had an N50 of 42,375,988 (*n* = 13), indicating the presence of nearly chromosome-length scaffolds. At face value, the scaffolding step seemed to decrease the overall quality of the assembly by adding gaps and increasing the number of scaffolds slightly. However, this step was necessary because the initial Hifiasm assembly did not contain chromosome-scale scaffolds, and the only way to obtain such scaffolds, given our sequencing strategy, was to scaffold against an existing assembly.

**Table 1.** Assembly stats results for the 2 existing chromosome-level genome assemblies for the *GRZ* strain of *N. furzeri* compared to our new assembly for strain *MZM-0403*.

Genome assembly	NCBI accession number(s)	Total length (Gb)	No. of scaffolds	N50	N90	*N* count (Mb)	Gaps
GRZ_2015	GCF_001465895.1, GCA_001465895.2	1.24	5,897	57,367,160 *n* = 9	152,787 *n* = 180	385.7	85,562
GRZ_2020	GCA_014300015.1	1.05	40,229	49,927,339 *n* = 9	34,229 *n* = 439	65.9	128,178
MZM-0403	GCA_027789165.1, GCF_027789165.1	1.51	2,681	42,375,988 *n* = 13	725,287 *n* = 147	102.6	3,093
MZM-0403 (before scaffolding)	NA	1.40	2,232	1,564,885 *n* = 247	390,456 *n* = 922	0	0

We also include the stats for the contig-level assembly of MZM-0403 before scaffolding. The GRZ genome assemblies are here referred to as GRZ_2015 (Reichwald *et al*. 2015) and GRZ_2020 (Willemsen *et al*. 2020). The total length of each assembly is the sum of all sequences present in the genome fasta file, here given in billions of base pairs (Gb). We also report the total number of scaffolds (or contigs in the case of MZM-0403 before scaffolding), the N50 and N90 values, the number of unknown base pairs in each assembly (represented by Ns in the sequences) given in millions of base pairs (Mb), and the number of gaps (i.e. runs of Ns). Note that these results include the mtDNA (in the MZM-0403 assembly, fasta record NC_011814.1 is mtDNA).

The annotation report can be accessed via NCBI (https://www.ncbi.nlm.nih.gov/refseq/annotation_euk/Nothobranchius_furzeri/GCF_027789165.1-RS_2023_03/). The annotation includes 36,805 genes and pseudogenes, of which 24,060 are protein-coding, 11,106 are noncoding, and 1,550 are pseudogenes. The annotation also includes 46,728 unique mRNA sequences and 46,821 coding sequences, indicating that many protein-coding genes have multiple isoforms represented in the annotation.

### Quality assessment and comparison to existing assemblies

The Assembly Stats reports for GRZ_2015, GRZ_2020, and our MZM-0403 assembly are shown in Table 1. One notable pattern is that our new MZM genome assembly is 1.51 Gb in total length, which is considerably larger than both GRZ assemblies and more similar to the expected genome size based on flow cytometry (Reichwald *et al*. 2009) and k-mer-based estimates (Reichwald *et al*. 2015). With only 2,681 contigs in the scaffolded assembly, this new MZM-0403 assembly is less fragmented than any of the previous *N. furzeri* assemblies. This reduced fragmentation is reflected in the dramatically reduced number of gaps in the MZM-0403 assembly, which has only 3,093 gaps compared to 85,562 and 128,178 gaps in the GRZ_2015 and GRZ_2020 assemblies, respectively. The total number of unknown bases in the MZM-0403 assembly is still quite large, with 102.6 million Ns in the scaffolded assembly (Table 1). Before scaffolding, the MZM-0403 assembly had no gaps, so these gaps were added to build chromosome-scale sequences scaffolded against GRZ_2015. In general, the MZM-0403 assembly has a relatively small number of relatively long gaps, whereas the other 2 assemblies have extremely numerous small gaps scattered across their chromosomes (Table 1).

The distribution of gaps relative to coding sequences can be important in terms of the utility of genome assemblies for various purposes. For instance, gaps in coding sequences may be problematic for evolutionary analyses or functional analyses of gene products. In addition, gaps near coding sequences can interfere with any application that requires knowledge of noncoding sequences near genes or within introns. For example, promoter sequences are often of interest for applications in molecular biology, and conserved noncoding elements can be important sources of data related to the evolution of gene regulatory networks.

To assess these *N. furzeri* assemblies in terms of the genomic distribution of gaps, we scanned each assembly for runs of Ns within 10,000 bp upstream or downstream of coding sequences, within exons, and within introns (Table 2). This analysis shows a dramatic difference among assemblies. Nearly two-thirds of annotated genes in the GRZ_2015 and GRZ_2020 assemblies have runs of Ns within 10 kb upstream of the gene, and we see the same pattern downstream of genes (Table 2). These values are reduced to only about 4% in the MZM-0403 assemblies. We see a similar result for introns, where genes in GRZ_2015 and GRZ_2020 have runs of Ns in at least one intron in approximately half the cases (Table 2). Again, in the MZM-0403 assembly, this number is around 4%. All of the assemblies were unlikely to have runs of Ns in the actual coding sequences (Table 2). In general, our new MZM-0403 assembly is much more likely to have complete information regarding flanking sequences and introns of genes compared to the previously available assemblies. This result is a consequence of the MZM-0403 assembly taking advantage of long-read sequencing technology, which tends to provide long stretches of sequence information that can span short repetitive regions in the vicinity of genes.

**Table 2.** Distribution of runs of Ns (i.e. gaps in the assembly) in the 3 *N. furzeri* genome assemblies near coding sequences.

Genome assembly	Number of annotated genes	No. genes with Ns within 10 kb upstream	No. genes with Ns within 10 kb downstream	No. genes with Ns in coding sequence	No. genes with Ns in introns
GRZ_2015	22,207	13,813 (62.2%)	13,346 (60.1%)	53 (0.2%)	10,329 (46.5%)
GRZ_2020	20,242	12,945 (64.0%)	12,755 (63.0%)	739 (3.7%)	10,075 (49.8%)
MZM-0403	24,060	929 (3.9%)	926 (3.8%)	0 (0%)	1,017 (4.2%)

The BUSCO results show that all of the *N. furzeri* genome assemblies under consideration here are very complete in terms of gene content (Table 3). The percentage of complete BUSCOs ranges from a low of 93.9% in the MZM-0403 assembly to a high of 95.7% in the GRZ_2020 assembly (Table 3). Both existing *N. furzeri* genomes based on the GRZ strains have slightly higher BUSCO scores compared to the MZM-0403 assembly. This difference is probably due primarily to 2 factors. First, our sequencing depth of ∼48 × coverage likely missed some small parts of the genome and the genes that reside therein. Second, both GRZ assemblies used RNA-seq data as part of their assembly strategy, and we did not. Although RNA-seq data were utilized during the annotation process, the MZM-0403 assembly was constructed exclusively using PacBio HiFi reads.

**Table 3.** BUSCO results for the three chromosome-level *N. furzeri* assemblies.

Genome	Complete BUSCOs	Complete (single copy)	Complete (duplicated)	Fragmented	Missing
GRZ_2015	3,460 (95.0%)	3,434	26	69 (1.9%)	111 (3.1%)
GRZ_2020	3,482 (95.7%)	3,411	71	52 (1.4%)	106 (2.9%)
MZM-0403	3,420 (93.9%)	3,379	41	53 (1.5%)	167 (4.6%)

The BUSCO analysis used the *Actinopterygii* odb10 database, which includes 3,640 genes that are expected to occur in all ray-finned fish genomes.

**Fig. 1.**
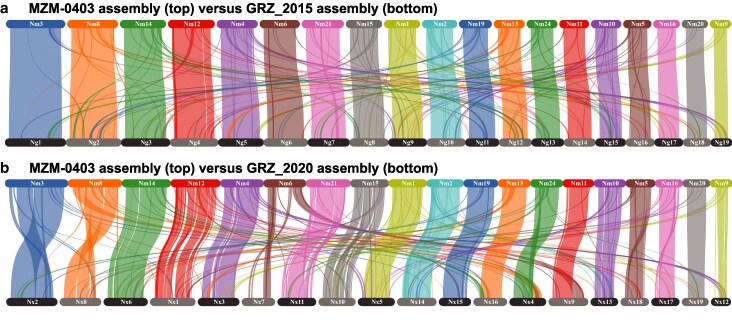
Synteny analysis comparing the MZM-0403 assembly to the existing GRZ genome assemblies, a) GRZ_2015, and b) GRZ_2020. In each comparison, the MZM-0403 assembly is on the top and the GRZ assembly is on the bottom. The 19 linkage groups correspond to the 19 chromosomes known to occur in the haploid *N. furzeri* genome. While the genome assemblies are mostly similar in terms of gene order, the MZM-0403 assembly is more similar to the GRZ_2015 assembly than it is to the GRZ_2020 assembly because the GRZ_2015 assembly was used for scaffolding in the present analysis. Chromosomes are represented by alphanumeric codes in this figure, and the corresponding fasta header identification numbers are shown in Table 4.

Our final comparison of the GRZ assemblies with the MZM-0403 assembly relied on synteny analysis. The comparisons of MZM-0403 with GRZ_2015 and with GRZ_2020 are shown in Fig. 1. Each fasta record in each assembly was assigned a code starting at the beginning of the fasta file containing the genome sequences. For GRZ_2015, the code was Ng, followed by the numerical position of the record in the fasta file, and for GRZ_2020 and MZM-0403, the code prefixes were Nx and Nm, respectively. We based chromosomal homology across assemblies on the number of shared genes in an all vs all blastp search. Table 4 lists the fasta header identification codes from each assembly, as well as our alphanumeric codes for each of the 19 *N. furzeri* chromosomes. We use the chromosome order established by the GRZ_2015 assembly. Table 4 also gives the length of each chromosome in each assembly. In general, the GRZ_2015 assembly has the longest chromosomes, followed by MZM-0403, and then by GRZ_2020. This pattern does not necessarily imply that GRZ_2015 is the highest quality assembly, however, as these chromosome lengths include a substantial number of unknown base pairs representing gaps in the assemblies. For example, the GRZ_2015 assembly includes 385.7 Mb of Ns, which comprise ∼31% of the assembly.

**Table 4.** List of homologous chromosomes across the GRZ_2015, GRZ_2020, and MZM-0403 assemblies.

Chromosome	GRZ_2015 Genome ID (Fig. 1 ID)	Length (Mb)	GRZ_2020 Genome ID (Fig. 1 ID)	Length (Mb)	MZM-0403 Genome ID (Fig. 1 ID)	Length (Mb)
1	NC_029649.1 (Ng1)	98.476	CM025009.1 (Nx2)	74.218	NW_026539916.1 (Nm3)	95.007
2	NC_029650.1 (Ng2)	88.342	CM025015.1 (Nx8)	57.431	NW_026539921.1 (Nm8)	79.553
3	NC_029651.1 (Ng3)	76.489	CM025013.1 (Nx6)	61.956	NW_026539927.1 (Nm14)	70.600
4	NC_029652.1 (Ng4)	75.031	CM025008.1 (Nx1)	64.217	NW_026539925.1 (Nm12)	72.159
5	NC_029653.1 (Ng5)	70.255	CM025010.1 (Nx3)	57.829	NW_026539917.1 (Nm4)	61.616
6	NC_029654.1 (Ng6)	67.275	CM025014.1 (Nx7)	43.217	NW_026539919.1 (Nm6)	59.176
7	NC_029655.1 (Ng7)	63.667	CM025018.1 (Nx11)	52.942	NW_026539934.1 (Nm21)	63.363
8	NC_029656.1 (Ng8)	57.680	CM025017.1 (Nx10)	49.826	NW_026539928.1 (Nm15)	52.548
9	NC_029657.1 (Ng9)	57.367	CM025012.1 (Nx5)	49.927	NW_026539914.1 (Nm1)	52.024
10	NC_029658.1 (Ng10)	57.198	CM025021.1 (Nx14)	54.027	NW_26539915.1 (Nm2)	50.306
11	NC_029659.1 (Ng11)	48.234	CM025022.1 (Nx15)	41.585	NW_026539932.1 (Nm19)	46.087
12	NC_029660.1 (Ng12)	46.077	CM025023.1 (Nx16)	44.056	NW_026539926.1 (Nm13)	42.478
13	NC_029661.1 (Ng13)	45.871	CM025011.1 (Nx4)	49.614	NW_026539937.1 (Nm24)	42.376
14	NC_029662.1 (Ng14)	44.252	CM025016.1 (Nx9)	54.464	NW_026539924.1 (Nm11)	40.257
15	NC_029663.1 (Ng15)	41.916	CM025020.1 (Nx13)	33.713	NW_026539923.1 (Nm10)	41.464
16	NC_029664.1 (Ng16)	39.628	CM025025.1 (Nx18)	35.076	NW_026539918.1 (Nm5)	37.393
17	NC_029665.1 (Ng17)	38.285	CM025024.1 (Nx17)	34.492	NW_026539929.1 (Nm16)	33.937
18	NC_029666.1 (Ng18)	37.194	CM025026.1 (Nx19)	32.272	NW_026539933.1 (Nm20)	33.747
19	NC_029667.1 (Ng19)	25.484	CM025019.1 (Nx12)	20.675	NW_026539922.1 (Nm9)	24.826

Here, we present the fasta header IDs for each *N. furzeri* chromosome as they appear in the 3 publicly available chromosome-level assemblies. Homology was determined by synteny, so chromosomes with the most shared genes are considered homologs for this table. We also show, in parentheses, the chromosomal ID that we used in Fig. 1. In each case, these IDs are numbered from the beginning of the genomic fasta file. For instance, chromosome 1 is the first record in the GRZ_2015 assembly (Ng1), the second record in the GRZ_2020 assembly (Nx2), and the third record in the MZM-0403 assembly (Nm3). We also show the length of each chromosome in megabases. Chromosomes number (left column) follows the convention established by GRZ_2015.

The synteny comparisons of MZM-0403 with GRZ_2015 and GRZ_2020 are shown in Fig. 1. Not surprisingly, the MZM-0403 genome assembly is almost entirely collinear with the GRZ_2015 assembly. This result was expected, as the GRZ_2015 assembly was used to scaffold the MZM-0403 assembly. In contrast, the comparison of MZM-0403 with GRZ_2020 does show some substantial structural differences. For instance, chromosome Nm6 (NW_026539919.1) in the MZM-0403 assembly is split onto 2 different chromosomes in the GRZ_2020 assembly (chromosomes Nx7 and Nx9; see Table 4 for their fasta ID numbers). These discrepancies among assemblies cannot be resolved by our analysis here. Rather, future work will be necessary to determine the true composition of each *N. furzeri* chromosome.

### Repeat content

Table 5 shows the results of the RepeatMasker analysis for the MZM-0403 genome assembly, as well as for the X-chromosome and Y-chromosome fragments, which will be discussed later. Nearly two-thirds of the MZM-0403 assembly is masked as repetitive DNA by RepeatMasker (Table 5). Interspersed elements are the primary source of repetitive sequences, and retro-elements followed by DNA transposons make the largest contributions to the interspersed element category. Many of these elements remain unclassified. Satellites and simple repeats are also important contributors to the repeat landscape of the MZM- 0403 assembly, with each accounting for ∼19% of the genomic sequence.

**Table 5.** RepeatMasker results for the MZM-0403 assembly and the X- and Y-chromosome fragments obtained from the phased Hifiasm assemblies.

Repeat type	MZM-0403 Number of elements	MZM-0403 Percentage of sequence	X-fragment Number of elements	X-fragment Percentage of sequence	Y-fragment Number of elements	Y-fragment Percentage of sequence
**Retroelements:**	725,242	14.07	1849	19.93	1379	11.82
SINES	94,908	0.87	298	1.14	218	0.70
LINEs	393,645	9.44	1237	14.57	921	8.32
LTR elements	236,689	3.76	314	4.22	240	2.79
**DNA transposons:**	386,994	7.13	1082	8.99	920	5.92
hobo-Activator	169,147	3.51	477	4.43	387	2.70
Tc1-IS630-Pogo	95,370	1.91	321	2.91	254	1.84
En-Spm	76	0.00	0	0.00	0	0
MULE-MuDR	6431	0.08	37	0.19	30	0.11
PiggyBac	5511	0.07	13	0.04	12	0.03
Tourist/Harbinger	10,975	0.19	36	0.24	36	0.17
Other	3200	0.07	6	0.02	6	0.01
**Rolling-circles:**	1986	0.03	0	0	18	0.16
**Unclassified:**	2,526,723	24.04	3973	23.06	4435	23.86
**Total interspersed:**		45.24		51.98		41.60
**Small RNA:**	30,941	0.20	53	0.16	42	0.09
**Satellites:**	948,644	18.69	118	4.71	704	30.65
**Simple repeats:**	1,282,376	19.06	1116	2.63	967	1.84
**Low complexity:**	20,766	0.09	78	0.14	61	0.08

The total length of the MZM-0403 assembly, excluding Ns, was 1,410,473,859 bp, and RepeatMasker masked 66.35% of the assembly. The X-fragment was 3,385,904 bp with 59.01% masked, and the Y-fragment was 4,453,144 bp with 73.95% masked. Neither of these latter sequences contained any Ns.

The assembled repeat landscape probably represents the biggest difference between our MZM-0403 assembly and the other *N. furzeri* assemblies. For instance, Reichwald *et al*. (2015) reported that 35% of the GRZ_2015 assembly consisted of repetitive DNA, which is a considerably smaller proportion than we observed for MZM-0403. Based on their Sanger sequencing data, however, they estimated that the genome was actually 64.6% repetitive DNA. This number agrees well with our results for MZM-0403, which had 66.35% of the assembly masked by RepeatMasker. Furthermore, Reichwald *et al*. (2015) used PacBio sequencing to confirm that most of the gaps in their assembly consisted of repeats. The MZM-0403 assembly's almost complete lack of gaps in the vicinity of coding sequences suggests that we successfully sequenced through most of these repetitive regions and included them in the assembly.

Despite our assembly arguably containing most of the repetitive sequences present in the *N. furzeri* genome, much of this repetitive DNA is not assembled onto the chromosomes. The sum total length of the 19 chromosomes in our assembly is 998.9 Mb, including gaps, which means that ∼500 Mb of the assembly occurs on 2,661 unplaced scaffolds. This situation is not surprising, because we used the GRZ_2015 assembly for scaffolding and many of these repetitive sequences were probably missing from that assembly. Nevertheless, there is substantial room for improvement in the future.

### Sex chromosomes

We were fortunate that the phased contigs produced by Hifiasm contained apparent fragments of the X- and Y-chromosomes. Our comparison of *gdf6* between the X- and Y-chromosome fragments recovers the expected differences between the female and male versions of *gdf6*, a finding that lends additional support to the notion that *gdf6* is the sex-determination locus. The *gdf6* sequence from MZM-0403 scaffold h1tg00029l is identical to the *gdf6* nucleotide sequence annotated in the GRZ_2015 assembly. Because the GRZ_2015 assembly was based on a female individual, we know that this version of *gdf6* occurs on the X-chromosome and can conclude that the h1tg00029l scaffold from the MZM assembly is a fragment of the MZM X-chromosome. On MZM scaffold h2tg000474l, we find another version of the *gdf6* locus, which differs substantially from the X-chromosomal version of this gene. In particular, the version of *gdf6* on h2tg000474l possesses the sequence features that have been observed for the Y-chromosomal version of *gdf6* (Reichwald *et al*. 2015; Štundlová *et al*. 2022). These features include a 9-bp deletion in the coding sequence and a 241-bp deletion in the 3′-UTR relative to the X-chromosomal version of *gdf6*. In addition, the Y-chromosomal *gdf6* coding sequence differs from the X-chromosomal version by ∼1.6% at the nucleotide level and ∼3.3% at the amino acid level (Table 6). From these observations, we can conclude that h2tg000474l does indeed represent a fragment of the *N. furzeri* Y-chromosome.

**Table 6.** Genes in the sex-chromosome region of the MZM-0403 assembly. The gene IDs from the genome annotations are shown in the first column.

Gene ID GRZ_2015 (MZM-0403)	GRZ_2015 chromosomal location (start–finish)	MZM-0403 chromosomal location (start–finish)	Nucleotide divergence GRZ_2015 vs MZM X	Nucleotide divergence GRZ_2015 vs MZM Y	Amino acid divergence GRZ_2015 vs MZM X	Amino acid divergence GRZ_2015 vs MZM Y
*ttk* (*ttk*)	37,475,897–37,486,487	32,869,733–32,881,272	0	0.00203	0	0.00204
*ebag9* (*ebag9*)	37,486,854–37,497,561	32,881,618–32,892,468	0	0.00156	0	0
*sybu* (*sybu*)	37,501,982–37,513,861	32,896,413–32,908,945	0	0.00247	0	0.00371
*gdf6* (*gdf6a*)	37,521,124–37,526,579	32,916,208–32,921,780	0	0.01595	0	0.03283
*eny2* (*eny2*)	37,718,820–37,721,232	33,114,892–33,117,290	0	0.00347	0	0
*trhr* (*trhra*)	37,930,468–37,945,156	33,259,888–33,274,954	0	0	0	0
*tmem7* (*LOC107378913*)	37,999,255–38,002,345	33,327,314–33,330,378	0	0.00229	0	0.00690
*emc2* (*LOC129163365*)	38,065,906–38,115,066	33,393,357–33,442,716	0	0	0	0
*eif3e* (*eif3e*)	38,122,450–38,153,585	33,450,163–33,508,387	0	0.00075	0	0
*rspo2* (*LOC129163366*)	38,193,062–38,261,500	33,517,901–33,597,488	0	0	0	0
*LOC107378918* (*LOC107378918*)	38,291,544–38,292,368	33,627,672–33,628,487	0	0.00709	0	0.01075
*LOC107378919* (*LOC107378919*)	38,295,708–38,296,406	33,631,912–33,632,591	0	0.00388	0	0.01176
*LOC107378920* (*LOC107378920*)	38,348,638–38,480,699	33,665,222–33,817,532	0.00007	0.00162	0.00022	0.00177
*LOC107378921* (*macf1b*)	38,488,541–38,529,898	33,825,723–33,866,521	0	0.00201	0	0.00352
*LOC107378924* (*zgc*)	38,538,581–38,544,535	33,875,082–33,881,660	0	0.00147	0	0.00221
*nfatc1* (*LOC107378925*)	38,580,724–38,648,941	33,915,750–33,985,645	0	0.00148	0	0.00222
*sall3* (*sall3a*)	39,025,098–39,039,988	34,217,840–34,232,742	0	0.00150	0	0.00075
*LOC107378931* (*NA*)	39,712,051–39,714,103	34,794,595–34,795,515	0	0	0	0
*LOC107378933* (*LOC107378933*)	39,776,840–39,794,064	34,857,820–34,876,271	0	0.00476	0	0
*znf236* (*LOC107378938*)	39,800,379–39,907,622	34,876,077–35,055,021	0	0.00244	0	0.00183

The next 2 columns show the region spanned by each gene in each assembly. In the GRZ_2015 assembly, NC_029653.1 is the sex chromosome, and in the MZM-0403 assembly, NW_026539917.1 is the sex chromosome. The last 4 columns show, for the coding sequence of each gene, the proportion of base pairs or amino acids that differ between the GRZ_2015 assembly and the putative X- and Y-chromosomes identified from MZM-0403. Note that LOC107378931 was not annotated in the MZM-0403 assembly.

**Fig. 2.**
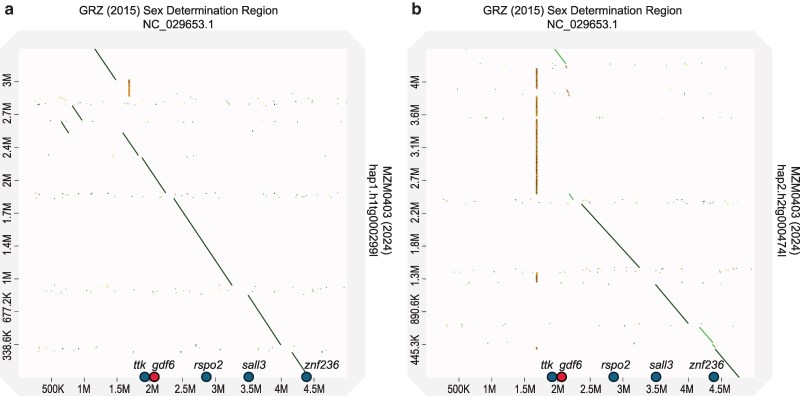
Dot plots comparing the putative X- and Y-chromosomal fragments identified in our assembly to the corresponding region from the GRZ_2015 genome assembly. The *x*-axis encompasses a region of about 5 million base pairs from chromosome 5 (NC_029653.1) of the GRZ_2015 assembly, starting at position 35,500,000 on the scaffold. The *y*-axes span the full length of a) the X-chromosomal contig and b) the Y-chromosomal contig from MZM-0403 assembled by Hifiasm. The approximate positions of some of the annotated genes from this region in the GRZ_2015 assembly are shown along the *x*-axis. Additional genes occur between *ttk* and *znf236*, and these are listed in order in Table 6. The putative sex-determination locus, *gdf6*, occurs around the 2 Mb mark on the *x*-axis. The regions of similarity proceed from the top left to the bottom right on these dot plots because the MZM-0403 contigs are reversed relative to the GRZ_2015 assembly.

We compared these X-chromosome and Y-chromosome fragments from MZM-0403 to the homologous region of the GRZ X-chromosome from the GRZ_2015 assembly using dot plots (Fig. 2). This analysis shows that the MZM-0403 X-chromosomal fragment is quite similar and largely collinear with the corresponding region of the GRZ_2015 X-chromosome (Fig. 2a; note that the sequences are reversed relative to one another in their respective assemblies). However, there are a few differences in assembly order, as evidenced by sequence fragments that are displaced from the main axis of similarity, especially in the upper-left corner of the dot plot. In addition, the MZM-0403 X-chromosome fragment contains several repetitive sequences that occur as gaps in the GRZ_2015 assembly. While the MZM-0403 X-chromosomal fragment is very similar to the homologous region of the GRZ_2015 assembly, we see a markedly different pattern for the MZM-0403 Y-chromosomal fragment (Fig. 2b). Notably, the MZM-0403 Y-chromosomal region is interrupted by a large stretch of repetitive sequences, ∼2 Mb in length. In addition, we observe regions with reduced sequence similarity, and a notable expansion of a second repetitive region (at the 1.3 Mb location, Fig. 2b).

The observed increase in repetitive sequences on the Y-chromosomal fragment is supported by our RepeatMasker analysis, which masked 59% of the MZM-0403 X-chromosome compared to 74% of the Y-chromosomal region (Table 5). This difference was driven primarily by an increase in the proportion of satellite sequences on the Y-chromosome (Table 5). The differences in repetitive sequence content between the X- and Y-chromosomes that we see in these relatively small fragments are consistent with the observation that *N. furzeri* has heteromorphic sex chromosomes (Štundlová *et al*. 2022), as such differences, extended to the scale of an entire chromosome, could easily result in chromosomes that differ in length. At present, however, the overall similarity of the X- and Y-chromosomes in *N. furzeri*, in terms of DNA sequence, makes the assembly of separate sex chromosomes virtually impossible with our current algorithms. Rather, when the whole-genome assembly is based on a male sample, we obtain a chimeric chromosome that has a mixture of features from the X- and Y-chromosomes.

A comparison of the gene content on the MZM-0403 X- and Y-chromosomal regions shows that all but one gene present in the GRZ_2015 annotation in this span of the X-chromosome are present on both the X- and Y-chromosomes of MZM-0403. A total of 21 genes are located in the GRZ_2015 X-chromosomal region that overlaps with the MZM-0403 X- and Y-chromosomal fragments. The one gene that is missing from the MZM-0403 annotation, as well as from the X- and Y-chromosomal contigs, is *LOC107378932* from the GRZ_2015 assembly. An inspection of this region of the MZM-0403 X- and Y-chromosomal sequences shows that the first exon of this gene is missing an ATG (methionine) start site. We also found that 3 of the 7 exons from the GRZ_2015 version of *LOC107378932* had no blast hits in the MZM-0403 genome. These results suggest that this locus has become pseudogenized in MZM-0403 and is in the process of being lost from the genome.

Sequence comparisons of the coding sequences of the remaining 20 genes that are present in this region of the GRZ_2015 and the MZM-0403 assembly show that the MZM-0403 X-chromosomal loci are almost always identical at the nucleotide and amino acid levels to the homologous GRZ_2015 loci (Table 6). The only exception is LOC107378920, which has only one nucleotide difference in its coding sequence. This difference turns out to be nonsynonymous, so this locus also has a single amino acid difference between the GRZ_2015 X-chromosomal sequence and the MZM-0403 X-chromosomal sequence.

We see a very different pattern for the MZM-0403 Y-chromosomal sequences, where almost every gene on the Y-chromosome shows at least some coding sequence divergence from the GRZ_2015 X-chromosomal sequence (and hence the MZM-0403 X-chromosomal sequence; Table 6). The most divergent gene is *gdf6*, which lends additional support to the notion that this gene is the causal sex-determination locus. Four genes (20%) show no divergence between the X- and Y-chromosomes. The remaining genes, excluding *gdf6*, display between 0.075% and 0.71% nucleotide sequence divergence between the X- and Y-chromosomes (Table 6). Amino acid divergence ranges from 0.075% to 1.18%. Even though these levels of divergence are relatively small, the observation that gene divergence is consistently higher between the MZM-0403 Y-chromosome and the GRZ_2015 X-chromosome than between the MZM-0403 X-chromosome and the GRZ_2015 X-chromosome implies that the Y-chromosome's nonrecombining region spans this entire region of the genome. The nonrecombining region likely extends even farther than the regions we have identified here, but a definitive investigation will require a better assembly of the Y.

### Conclusions

This study provides a novel genetic resource to facilitate research involving *N. furzeri*. Importantly, we provide a whole-genome, chromosome-level assembly of the interesting *N. furzeri MZM-0403* strain. This assembly should facilitate work aimed at understanding the genetic basis of phenotypic differences between *N. furzeri* populations. In particular, lifespan and male-specific traits differ substantially between MZM-0403 and GRZ, the strain for which genome assemblies were already available. In addition, we show that a whole-genome assembly based on PacBio HiFi reads has several advantages over assemblies based on short reads. The most important advantage is that PacBio sequencing can sequence through repetitive sequences, resulting in an assembly with fewer gaps. Another advantage is that extremely long contigs can be produced from HiFi assemblies alone. In addition, these HiFi-based assemblies can result in phased contigs, and this feature allowed us to recover X- and Y-chromosome fragments ∼3.5–4.5 Mb in length. Analysis of these fragments show that the Y-chromosome has a proliferation of repetitive sequences and that the nonrecombining region probably encompasses at least this entire portion of the sex chromosomes. The results of our study suggest that future work should endeavor to use PacBio HiFi sequencing technology to improve the assembly of the *N. furzeri* GRZ genome. Ideally, this technology would be combined with a method that provides positional information, such as Hi-C sequencing, to resolve the chromosome-level structural differences that are present in the current GRZ genome assemblies. In addition, the combination of PacBio HiFi sequencing and Hi-C library sequencing has great promise for studies of comparative genomics, which could be profitably applied to variation across *N. furzeri* strains as well as to variation among species within the genus *Nothobranchius*.

## Data Availability

The raw sequencing reads, genome assembly, and annotation can be obtained from NCBI, under BioProject PRJNA891775. The genome assembly RefSeq ID is GCF_027789165.1, and the GenBank ID is GCA_027789165.1. The accession number for the raw PacBio HiFi reads is SRR32095763.
